# Is quality of life associated with compliance to pharmacoterapy in patients with chronic kidney disease undergoing maintenance hemodialysis?

**DOI:** 10.1590/S1679-45082018AO4036

**Published:** 2018-04-06

**Authors:** Keila Batista Alves, Nathália Vital Guilarducci, Thiago dos Reis Santos, André Oliveira Baldoni, Alba Otoni, Sérgio Wyton Lima Pinto, Camila Zanette, Cristina Sanches

**Affiliations:** 1 Universidade Federal de São João del-Rei, Divinópolis, MG, Brazil; 2 Hospital São João de Deus, Divinópolis, MG, Brazil; 3 University of California, Irvine, CA, United States

**Keywords:** Renal insufficiency, chronic, Medication adherence, Quality of life, Renal dialysis, Insuficiência renal crônica, Adesão à medicação, Qualidade de vida, Diálise renal

## Abstract

**Objective:**

To analyzed the association of quality of life and compliance to drug treatment in chronic kidney disease patients.

**Methods:**

The Short Form Health Survey was used to evaluate the quality of life of these patients, and the therapeutic complexity index was verified. The Morisky-Green test and the Brief Medication Questionnaire were applied to check compliance to drug therapy.

**Results:**

A total of 197 patients were included. The Morisky-Green test and Brief Medication Questionnaire showed that most patients had low compliance to treatment (50.3% and 80.6%, respectively). Compliance was highly associated with gender (male) and slightly associated with complexity of therapy, mental health, and social aspects.

**Conclusion:**

We observed a slight association between compliance to pharmacotherapy and quality of life and complexity of therapy, and a strong association with gender.

## RESUMO

**Objetivo:**

Analisar a associação entre a qualidade de vida e a adesão ao tratamento farmacológico de pacientes com doença renal crônica.

**Métodos:**

Utilizou-se o *Short Form Health Survey* para avaliar a qualidade de vida destes pacientes. O índice de complexidade terapêutico foi avaliado. O teste de Morisky-Green e o *Brief Medication Questionnaire* foram aplicados para avaliar a adesão a farmacoterapia.

**Resultados:**

Nos 197 pacientes que participaram do estudo, o teste de Morisky-Green e o *Brief Medication Questionnaire* mostraram que a maioria deles apresentava baixa adesão ao tratamento (50,3% e 80,6%, respectivamente). A adesão esteve altamente associada ao sexo (masculino) e ligeiramente associada à complexidade da terapia, à saúde mental e aos aspectos sociais.

**Conclusão:**

Observaram-se ligeira associação da adesão farmacoterapêutica com qualidade de vida e complexidade terapêutica, e forte associação com o sexo.

## INTRODUCTION

Chronic kidney disease (CKD) is a worldwide public health problem due to its increased prevalence, high costs, and mortality rate.^(^ ^1^ ^,^ ^2^ ^)^ According to the Brazilian Society of Nephrology (SBN) census, the estimated total number of patients under dialytic treatment in Brazil, in 2015, was 111,303, and 90% of them were treated free of charge by the Unified Health System (SUS – *Sistema Único de Saúde*).^(^ ^3^ ^)^

Patients on dialysis deal with a painful long-term treatment and its complications, which impacts their quality of life and that of their families.^(^ ^4^ ^–^ ^6^ ^)^

Adding to the abovementioned factors, it is expected that high number of comorbidities and, consequently, a high therapeutic complexity have an important impact on compliance.^(^ ^7^ ^)^

Regarding drug therapy, some factors may hinder compliance of these patients, such as therapeutic complexity, adverse reactions induced by the drugs, and lack of understanding regarding the prescribed therapy.^(^ ^8^ ^)^ Therefore, it is imperative to evaluate the impact on quality of life and therapeutic complexity index (TCI) on compliance to pharmacotherapy to assure drug efficacy and improve outcome in CKD patients.

## OBJECTIVE

To evaluate the association between quality of life of chronic kidney disease patients, therapeutic complexity index, and compliance to pharmacotherapy.

## METHODS

This cross-sectional study was developed in a philanthropic hospital located in the state of Minas Gerais, Brazil. Ethical approval was obtained (641.045/2014, CAAE: 30249814.1.0000.5545), and an Informed Consent was obtained from all individual participants enrolled in the study. A total of 209 end-stage renal disease patients (>18 years old) on hemodialysis were eligible for inclusion. We invited these patients to participate; 22 refused and 197 patients were enrolled. Data collection was carried out from August to November of 2014.

The interviews were performed individually in the hemodialysis room after the patients signing the Informed Consent Form. Each interview lasted, in average, 30 minutes.

The socioeconomic and demographic variables were gender, ethnicity, age, marital status, schooling level, comorbidities, private health insurance, transportations, household income (using the Brazilian minimum monthly wage of 2014 as basis, US$ 180.00), and employment situation.

The 36-Item Short Form Health Survey (SF-36), version 2, validated for Brazilian Portuguese was used to evaluate the quality of life of the CKD patients. This form has 36 items measuring eight scales of lifestyle: vitality (VIT), physical functioning (PF), bodily pain (BP), general health perceptions (GHP), physical role functioning (PRF), emotional role functioning (ERF), social role functioning (SRF), and mental health (MH). The questionnaire portrays a score from 0 to 100, in which 0 corresponds to the worst health status, and 100 to the best health status.^(^ ^9^ ^)^

In order to evaluate compliance to drug therapy, two questionnaires were used: the four item Morisky-Green Test (MGT) and the Brief Medication Questionnaire (BMQ).^(^ ^10^ ^)^ The analysis was performed for 196 out of 197 patients, since one patient was not on any medication. The MGT measures compliance of patients through four questions with dichotomous answers (“yes” and “no”). “No” is considered the expected answer for patients with good compliance; thus if there were no “yes” answer (positive), patients were included in the Compliance Group, and Non-Compliance was considered one to four positive answers.^(^ ^10^ ^)^ On the other hand, BMQ evaluates compliance to the drug therapy through three domains that identify barriers to compliance to drug regimen (seven questions), beliefs (two questions), and recall of medication treatment (two questions). Scores ≥1 indicate a potential non-compleinace in any domain. Compliance was classified as high (no positive answer), probable (one positive answer), probable low compliance (two positive answers) and low (three or more positive answers) in any domain.^(^ ^10^ ^)^

The pharmacotherapeutic profile showed that it is possible to verify the presence of polypharmacy and to determine the TCI. The presence of polypharmacy was evaluated, considered as the simultaneous usage of five or more medicines.^(^ ^11^ ^)^ The TCI was calculated according to Acurcio et al.,^(^ ^12^ ^)^ including measurements of number of medicines, frequency, and type of actions required for self-administration. The TCI was calculated considering only medication regimens dependent on patient action. Thus medicines used during and/or after dialysis sessions were disregarded. One patient was excluded from the calculation since he was not on any medicine.

### Statistical analysis

Statistical analysis was performed using the Statistical Package for the Social Sciences (SPSS), version 16.0 software. Normality was tested by the Kolmogorov-Smirnov test. The Mann-Whitney test was used to evaluate the difference between the questionnaire domains of the SF-36 and the gender variables, MGT and each domain of the BMQ (regimen, belief, and memory), and the TCI with MGT and BMQ. The established significance level was 5% (p<0.05).

The odds ratio (OR) was estimated using compliance as outcome, with the respective 95% of confidence interval (95%CI) using logistic regression. We included in the multivariate model the variables with p<0.2 for the univariate analysis, and those with plausibility to be inserted according to the literature. The stepwise backward method was used in order to insert the variables, and only the significant variables with p<0.5 remained. The explanatory variables used in the multivariate model were age, gender, race, number of comorbidities, hemodialysis time, functional capability, therapeutic complexity index, general state, mental health, pain, vitality, social role functioning, emotional role functioning, and physical role functioning.

## RESULTS

The majority of the population of the study was male (61.4%); the mean age was 56.5±13.2 years; 72.1% were retired due to disability, and 66.5% with monthly family income below two Brazilian minimum wages. Only 33.0% had private health insurance (Table 1). The transportation ways used were bus (60.9%), own vehicle (33.0%), ambulance (2.0%), walking (2.0%), taxi (1.0%) or no answer (02; 1.0%). The family income, based on monthly minimum wages, was below two for 66.5%, between two and five for 9.6%, above five minimum wages for 1.5%, and 22.0% did not know.

**Table 1:** Socioeconomic and demographic characteristics of a population on dialysis

Characteristics		n (%)
Male gender		121 (61.4)
Age, years		
	25-48	54 (27.4)
	49-56	45 (22.8)
	57-66	53 (26.9)
	≥67	45 (22.8)
Ethnicity		
	White	142 (72.1)
	Black	55 (27.9)
Marital status		
	Married	108 (54.8)
	Single	57 (28.9)
	Widow/widowed	26 (13.2)
	Divorced	6 (3.0)
Retired		
	Yes	142 (72.1)
	No	55 (27.9)
Schooling level		
	Elementary school, incomplete	113 (57.3)
	Elementary school, complete	35 (17.8)
	High school, complete	32 (16.2)
	Further education	10 (5.1)
	Illiterate	5 (2.5)
	No answer	2 (1.0)

The main comorbidities reported were systemic arterial hypertension (80.2%), *diabetes mellitus* (29.9%), congestive heart failure (24.4%), ulcer/gastritis (10.2%), depression (8.6%), bone diseases (6.1%), and neoplasia (0.5%).

For quality of life, measured according to the SF-36, the domains with lowest average scores were PRF (0.0) and ERF (33.3). On the other hand, the domain SRF showed the best average score (100.0). Female patients demonstrated lower quality of life scores than males in most domains, according to table 2.

**Table 2:** Values yielded from domains of the 36-Item Short Form Health Survey and comparison with the gender variable

Domains	Total value	Women (n=76)	Men (n=121)	p value*
Physical functioning	60 (25-80)	47.5 (20-71.3)	70 (40-85)	0.002
Physical role functioning	0 (0-50)	0 (0-50)	25 (0-50)	0.071
Bodily pain	62 (22-100)	41 (22-100)	100 (30-100)	0.013
General health perceptions	55 (45-67)	52(40-62.8)	57 (50-67)	0.012
Vitality	65 (55-75)	60 (55-75)	65 (55-75)	0.033
Social role functioning	100 (62.5-100)	100 (62.5-100)	100 (62.5-100)	0.412
Emotional role functioning	33.3 (33.3-100)	33.3 (33.3-100)	33.3 (33.3-100)	0.766
Mental health	76 (60-92)	72 (56-89)	80 (74-96)	0.017

Data expressed in medians (Quartiles 25-75%).

* Statistical test: Mann Whitney.

Regarding the variable polypharmacy 65.9% of patients took more than five medicines, and no significant difference with SF-36 domains was noticed (p>0.05). Compliance to pharmacotherapy as per MGT showed that 50.3% of patients complied with. When associating with the SF-36 domains, a significant difference was noticed, as shown on table 3.

**Table 3:** Comparison of compliance to pharmacotherapy by the Morisky-Green Test with the 36-Item Short Form Health Survey domains

Domains	Compliance (n=99)	Non-compliance (n=98)	p value*
Physical functioning	70 (0-100)	50 (5-100)	0.011
Physical role functioning	25 (0-100)	0 (0-100)	0.014
Bodily pain	100 (0-125)	31.50 (0-100)	<0.001
General health perceptions	62 (20-134)	45 (20-80)	<0.001
Vitality	70 (25-375)	55 (15-325)	<0.001
Social role functioning	100 (25-100)	62.5 (13-113)	<0.001
Emotional role functioning	33.33 (0-100)	33.33 (0-100)	0.501
Mental health	88 (20-100)	60 (8-100)	<0.001

Data expressed in median (Quartiles 25-75%).

* Statistical test: Mann-Whitney.

Evaluating compliance through the BMQ, the final score showed that 80.6% of patients achieved scores related to potential low compliance. In the regimen domain, 92.2% of patients showed positive potential for non-compliance (score ≥1). In the belief domain, only 12.8% of patients presented positive potential for non-compliance (score ≥1). In the “patient recall of medicine treatment” domain, which analysed a scheme of multiple doses and the difficulty of patients to remember taking their medications, 96.9% of patients showed potential non-compliance (score ≥1). However, “regimen” and “recall/memory” domains were more closely related to the potential low compliance to drug therapy. A significant difference relating BMQ and quality of life was observed, as is shown on table 4.

**Table 4:** Comparison between compliance to pharmacotherapy, according to Brief Medication Questionnaire (BMQ), and the do 36-Item Short Form Health Survey (SF-36) domains

SF-36 domains	BMQ score =0 (compliance)	BMQ score ≥1 (non-compliance)	p value*
	**BMQ – regimen domain**		
	**n=5**	**n=192**	
Physical functioning	85 (70-90)	55 (25-80)	0.006
Physical role functioning	50 (25-75)	0 (0-50)	0.061
Bodily pain	100 (61-100)	61 (22-100)	0.134
General health perceptions	62 (60-70)	55 (45-67)	0.046
Vitality	75 65-75)	65 (55-75)	0.164
Social role functioning	75 (62.5-100)	100 (62.5-100)	0.862
Emotional role functioning	66.7 (33.3-100)	33.3 (33.3-100)	0.909
Mental health	76 (64-100)	76 (60-92)	0.449
	**BMQ – belief domain**		
	**n=172**	**n=25**	
Physical functioning	60 (25-55)	60 (20-80)	0.804
Physical role functioning	0 (0-50)	0 (0-25)	0.186
Bodily pain	72 (22-100)	32 (22-100)	0.055
General health perceptions	55 (45-67)	52 (40-60)	0.082
Vitality	65 (55-75)	55 (45-75)	0.035
Social role functioning	100 (62.5-100)	62.5 (62.5-100)	0.004
Emotional role functioning	33.3 (33.3-100)	33.3 (0-66.7)	0.105
Mental health	80 (64-96)	56 (44-72)	<0.0001
	**BMQ – memory domain**		
	**n=7**	**n=190**	
Physical functioning	45 (32.5-68.8)	60 (25-80)	0.330
Physical role functioning	0 (0-37.5)	0 (0-50)	0.466
Bodily pain	100 (71.5-100)	61 (22-100)	0.332
General health perceptions	62.5 (54-66.5)	55 (45-67)	0.590
Vitality	67.5 (56.3-75)	65 55-75)	0.894
Social role functioning	81.3 (62.5-100)	100 (62.5-100)	0.777
Emotional role functioning	100 (25-100)	33.3 (33.3-100)	0.843
Mental health	68 (60-82)	76 (60-92)	0.391
Saúde mental	68 (60-82)	76 (60-92)	0,391

Data expressed as medians (25th to 75th quartiles).

* Statistical test: Mann Whitney.

The TCI was compared with the compliance profile and no significant difference (p=0.953) was obtained for the MGT test, with median values of 15 (0-34), for compliance, and 15 (0-29), for non-compliance. The same result was observed for BMQ beliefs, in which the Compliance Group presented a TCI of 14 (0-34) and the Non-Compliance Group, of 16 (6-25), with p=0.422. For regimen and memory domains, a significant difference was obtained. For the regimen domain, the Compliance Group showed lower TCI levels than the Non-Compliance Group, 9 (0-32) and 14.5 (4-27), respectively (p=0.04), and for the memory domain, 5 (0-7) for the Compliance Group *versus* 15 (2-34) for the Non-Compliance Group (p<0.001).

Finally, table 5 presents the logistic regression analysis results, showing the association between BMQ regimen *versus* TCI (OR=0.856) and age (OR=0.934); BMQ beliefs *versus* SRF (OR=1.021) and MH (OR=1.029); and BMQ-recall *versus* male sex (OR=9.354) and TCI (OR=0.524), as well as an association between MGT and general health perception (OR=1.026), MH (OR=1.026) and TCI (OR=1.053).

**Table 5:** Quality of life factors associated with compliance to drug therapy

Categories		p value*	OR	95%CI
MGT compliant				
	General health perceptions	0.011	1.026	1.006-1.047
	Mental health	0.001	1.026	1.011-1.041
	TCI	0.019	1.053	1.008-1.101
BMQ regimen				
	TCI	0.004	0.856	0.771-0.952
	Age	0.005	0.934	0.891-0.980
BMQ beliefs				
	Social role functioning	0.014	1.021	1.004-1.038
	Mental health	0.004	1.029	1.009-1.049
BMQ recall				
Gender				
	Female	-	1.00	-
	Male	0.047	9.354	1.027-85.232
TCI		0.004	0.524	0.337- 0.816

In the multivariate model, age, sex, race, number of comorbidities, hemodialysis time, functional capability, therapeutic complexity index, general state, mental health, pain, vitality, social functioning, emotional functioning, and physical functioning. Reference variable for *Brief Medication Questionnaire*: compliance.

* χ^2^ test. OR: odds ratio; 95%CI: 95% of confidence interval; MGT: Morisky-Green test; TCI: therapeutic complexity index; BMQ: Brief Medication Questionnaire.

## DISCUSSION

Until the present, the association of quality of life with compliance to pharmacotherapy has been poorly reported, despite the value of this information in the end-stage renal disease population.^(^ ^13^ ^)^ The relation between quality of life and treatment compliance has an important impact on the healthcare system, since non-compliance leads to an increase of new cases of hospitalizations, generating costs for this system.^(^ ^14^ ^)^

In the present study, compliance to pharmacotherapy by MGT showed that half of the patients had high compliance, while controversially, by BMQ, the final score showed 80.6% of potentially low compliance. Comparing compliance with quality of life scores, both general state and MH of the quality of life and TCI scores slightly increased the chance of compliance by MGT. Moreover, the compliance measurement using the BMQ (regimen domain) showed that the increased age and the TCI reduced the chance of compliance. Regarding the belief domain, the increase of SRF and MH values showed a slight increase in the chance of compliance. On the other hand, regarding the memory domain, being female and having higher TCI scores were highly associated with non-compliance. This result indicates that despite the association between quality of life and compliance, the main factors associated with compliance, especially recall, are the TCI and female sex. This result indicates that the perception of patients regarding their own health, SRF, and MH have a slight association with treatment compliance. Currently, data on patients undergoing hemodialysis are rare in the literature. A recent systematic review identified a positive relation between therapeutic compliance and quality of life in diabetic patients, which suggests that health professionals should take social aspects into consideration in order to have better clinical outcomes.^(^ ^15^ ^)^ Similar results were found for hypertensive patients regarding SRF and MH.^(^ ^16^ ^)^

DeOreo et al., found that patients who missed approximately two dialysis treatments per month, that is, who did not comply with the non-pharmacological treatment, showed better scores for mental and physical health, which demonstrates that when the patient is feeling mentally and physically well, he or she tends to skip the hemodialysis sessions.^(^ ^13^ ^)^ According to our results in which we evaluate the compliance to treatment with oral drugs, the patient tends to comply more to the pharmacological treatment when showing the same conditions.

The negative association between compliance and TCI in this study has been shown previously in a systematic review by Ghimere et al., in hemodialysis patients.^(^ ^17^ ^)^ Before addressing complexity, it is important to relate negative attitudes of patients regarding pharmacotherapy.^(^ ^18^ ^)^ In the present study, these attitudes might be related mainly to the belief domain of compliance, and be explained by poor quality of life of the patients over MH and SRF.

Comparing the quality of life scores with the variable sex, females showed more commitment in all domains and the recall domain was strongly associated with the compliance profile. Physiological changes, such as hypothalamic and ovarian dysfunction caused by the disease and intensified by hemodialysis, made females more limited in their daily activities with a low perception as to their general health status.^(^ ^19^ ^)^ This result contradicts the data obtained in previous studies, in which no significant difference was noted in any dimension of the SF-36 regarding the variable sex^(^ ^20^ ^)^ or shown in the male gender as a disadvantage for better quality of life scores.^(^ ^21^ ^)^

Regarding the drug therapy compliance profile analyzed by MGT and BMQ, the results obtained from these instruments were contradictory. Using MGT, half of the patients presented compliance profile, while using the BMQ, the majority of patients presented with a potential low compliance to the treatment. This divergence confirms the hypothesis that there is no gold standard for measuring compliance to pharmacological treatment.^(^ ^14^ ^,^ ^22^ ^,^ ^23^ ^)^

The population demographic and social data in this study were similar to the Brazilian Dialysis Census and other national studies, showing the prevalence of males, low educational level, and monthly income below two minimum wages.^(^ ^1^ ^,^ ^3^ ^,^ ^24^ ^–^ ^27^ ^)^ The low monthly income reported by patients may be related to retirement income and government financial aid for diseases, since half of the study population was at working age, which explained retirement caused by limitations from the disease. As expected, the higher prevalent diseases (hypertension and *diabetes mellitus*) have been the major causes of CKD development.^(^ ^28^ ^,^ ^29^ ^)^

Considering the quality of life scores, the results pointed out some problems mainly regarding PF, showing that physical status affects the performance of daily and work activities in CKD patients. Similar results were also found in another Brazilian study.^(^ ^30^ ^)^ However, Mortari et al.,^(^ ^31^ ^)^ found low scores for the PF limitations and for the GHP domain. Another domain that showed a high impact was ERF. This result was similar to other data in the literature, in which the most affected domains were PRF and ERF.^(^ ^32^ ^)^ Then again, in this study, the domain SRF had the maximum score and showed the best score, indicating that physical health or emotional problems do not interfere in the social life of end-stage renal disease patients.

The extreme values can be justified according to the type of variable that is obtained through SF-36, which is a categorical variable and does not allow a large diversity of intermediary values, or those close to “floor” and “ceiling”. Moreover, these results highlighted that the population is heterogeneous in relation to quality of life. This can be explained according to the broad social and economic diversity of the studied population, in addition to different phases and time of treatment.

Finally, the use of indirect methods of assessing compliance is assumed as a limitation, since it may present recall and information bias. Additionally, the cross-sectional design does not establish causality. Therefore, new longitudinal studies should be designed to evaluate the impact of quality of life on treatment compliance, so that interventional strategies are established in nephrology services, improving quality of life and consequently, compliance to treatment.

## CONCLUSION

Non-compliance to drug therapy may be associated with worse quality of life and high therapeutic complexity. Beyond improving therapeutic complexity, strategies should be based on mental health and social role functioning to improve compliance.
